# The Aftermath of Long-Term Cigarette Smoking on Telomere Length and Mitochondrial DNA Copy Number in Human Cumulus Cells Prior to In Vitro Fertilization—A Pilot Study

**DOI:** 10.3390/antiox11091841

**Published:** 2022-09-19

**Authors:** Fani Konstantinidou, Maria Cristina Budani, Guya Diletta Marconi, Francesca Gonnella, Annalina Sarra, Oriana Trubiani, Liborio Stuppia, Gian Mario Tiboni, Valentina Gatta

**Affiliations:** 1 Department of Psychological Health and Territorial Sciences, School of Medicine and Health Sciences, “G. d’Annunzio” University of Chieti-Pescara, 66100 Chieti, Italy; 2 Unit of Molecular Genetics, Center for Advanced Studies and Technology (CAST), “G. d’Annunzio” University of Chieti-Pescara, 66100 Chieti, Italy; 3 Faculty of Bioscience, Agri-Food and Environmental Technologies, University of Teramo, 64100 Teramo, Italy; 4 Department of Medical, Oral and Biotechnological Sciences, “G. d’Annunzio” University of Chieti-Pescara, 66100 Chieti, Italy; 5 Department of Philosophical, Pedagogical and Quantitative Economic Sciences, “G. d’Annunzio” University of Chieti-Pescara, 66100 Chieti, Italy; 6 Department of Innovative Technologies in Medicine & Dentistry, “G. d’Annunzio” University of Chieti-Pescara, 66100 Chieti, Italy

**Keywords:** cigarette smoke, telomere length, mitochondrial DNA copy number variation, oxidative stress, cumulus cells, gene expression, infertility

## Abstract

Cigarette smoking among women of reproductive age is known to take a toll on systemic health and fertility potential by severely impacting ovarian tissues and cells, such as granulosa and cumulus cells (CCs). The purpose of this study was to determine the potential damage caused by tobacco smoke at a molecular level in the CCs of females who had undergone in vitro fertilization. The level of intracellular damage was determined by estimating the average telomere length (TL) and mitochondrial DNA copy number (mtDNA-CN), as well as the expression profile of telomere maintenance genes *TERF1*, *TERF2*, *POT1* and microRNAs miR-155, miR-23a and miR-185. Western blotting analysis was performed to detect consequent protein levels of TERF1, TERF2 and POT1. Our results evidenced significantly lower relative TL and mtDNA-CN and a down-regulation pattern for all three described genes and corresponding proteins in the CCs of smokers compared with controls (*p* < 0.05). No significant differences were found in the miRNAs’ modulation. Combined, our data add another piece to the puzzle of the complex regulatory molecular networks controlling the general effects of tobacco smoke in CCs. This pilot study extends the until now modest number of studies simultaneously investigating the mtDNA-CN and TL pathways in the human CCs of smoking women.

## 1. Introduction

Cigarette smoking has become throughout the years a widespread and prevalent habit, constituting a considerable public health issue worldwide and representing the primary cause of millions of deaths in the past few decades alone [1]. In addition, it is a known risk factor of multiple pathologies, such as cardiovascular, respiratory and metabolic diseases and premature death [2], but also reproductive-related diseases [3]. A prevalence of cigarette smoking has been noticed among reproductive-aged women [4] as well as prior to and during pregnancy, potentially contributing to the manifestation of adverse health effects in smokers and their offspring [5]. The female reproductive system has been specifically shown to represent a sensitive target of tobacco smoke constituents, with ovaries having been demonstrated to be highly vulnerable to its noxious effects [6]. Steroidogenesis and folliculogenesis are the ovarian processes that appear to be most affected by this exposure [7], with consequent impacts on ovarian tissues and cells such as granulosa and cumulus cells (CCs). The paracrine interactions between the oocyte and surrounding granulosa cells are critical for optimal oocyte quality and embryonic development during folliculogenesis, making them vital for its correct growth and development [8]. More specifically, exposure to the harmful chemicals in cigarettes may overall contribute to subfertility and premature ovarian failure [9] as well as induce alterations in luteal steroidogenesis; follicular loss and apoptosis; oxidative stress (OS) and related gene expression [10]; autophagy; and irreparable damage to the telomeres, mitochondrial DNA (mtDNA), and mtDNA copy number (mtDNA-CN) [11].

Telomeres are highly specialized ribonucleoprotein structures at the ends of linear chromosomes that are essential for genome stability [12]. Human telomeres are known to shorten following each cell division, and as they steadily become critically short, cells no longer proliferate and become senescent. For this reason, telomere length (TL) has long been regarded as a marker of cellular aging [13]. It has been well established that cigarette smoking is a risk factor for aging-related diseases, but its association with biological aging and TL remains to this day not entirely elucidated. A possible suggested mechanism is that following cigarette smoking, free radicals may induce OS and inflammation, resulting in significantly shortened telomeres and causing cellular senescence and apoptosis [14].

Mitochondria are essential organelles able to produce energy and exist in a dynamic, interlinked network that is constantly reshaped by a closely regulated balance between two opposite processes, called fission and fusion, in order to maintain the mitochondrial content in all daughter cells and allow repair where mitochondrial damage is present [15]. They are highly sensitive to environmental toxicants, as well as tobacco smoke and air pollution individual constituents. Exposure to environmental toxicants can induce changes in mitochondrial respiration and metabolism, mtDNA damage and mtDNA-CN, elimination of dysfunctional mitochondria through mitophagy apoptosis and reduction-oxidation (redox) signaling. Tobacco smoke exposure induces cardiac mitochondrial damage and dysfunction. Several studies have shown that the mtDNA is in fact susceptible to oxidant/toxicant-derived damage through formation of strand breaks and thymidine dimers [11], as well as mtDNA copy number variation (mtDNA CNV) [16]. Regarding reproductive and developmental effects, tobacco smoke-induced alterations in mitochondrial function have been found able to contribute to growth restriction in utero and consequent low weight at birth, and other developmental outcomes [11].

The purpose of this study was to determine the damage to genomic and mitochondrial DNA caused by tobacco smoke in human cumulus cells of females who had undergone in vitro fertilization. The level of damage in isolated cumulus cells was detected by chromosome TL and related genes, microRNA, and protein expression, as well as by mtDNA CNV analysis, in an effort to determine possible correlations between chronic tobacco smoke exposure and aging-related conditions in human female reproductive cells.

## 2. Materials and Methods

### 2.1. Ethical Approval

The Institutional Review Board (Ethical Committee of University of Chieti-Pescara) approved the study with the document n°05, 8 March 2018.

### 2.2. Source of Cumulus Cells and Patients’ Selection Criteria

The CCs used for the purpose of the present study were donated with written informed consent by women undergoing assisted reproductive techniques (ART) at the ART Unit of the General Hospital G. Bernabeo of Ortona (Chieti, Italy). CCs were isolated from 5 smoking and 5 non-smoking (controls) women responding to the following criteria: age ≤ 39 years, normal karyotype, presence of both ovaries, regular ovulatory cycles, infertility due to tubal or male factors, body mass index (BMI) of 19–25 kg/m^2^, AMH level between 0.9 e 9 ng/mL, and basal FSH < 15 mIU/mL.

Contrarily, patients with grade III or IV endometriosis according to the American Society for Reproductive Medicine (ASRM), with history of poor response in previous ART cycles (≤3 oocytes retrieved) or with history of severe ovarian hyperstimulation syndrome (OHSS) were excluded. In addition, patients with antral follicle count (AFC) ≤ 7 or with endocrinopathies together with those using micronutrient supplements or with a number of ≥3 ART attempts without a clinical pregnancy were excluded from the study.

Cigarette smoking was assessed with the use of a questionnaire.

### 2.3. Ovarian Stimulation Protocols

Controlled ovarian stimulation (COS) was achieved with the exogenous administration of gonadotropins (recombinant follicle stimulating hormone (rFSH) or follitropin alpha biosimilars) starting from the second/third day of the menstrual cycle. The gonadotropin doses were adjusted on the basis of the patient’s ovarian response from stimulation day 5 until the day of human chorionic gonadotropin (hCG) administration.

In order to suppress the luteinizing hormone (LH) surge and according to the flexible protocol with multi-dose injections, daily injections of GnRH antagonist began when at least 1 follicle reached an average diameter ≥ 14 mm and continued until the day of hCG, administered when at least 3 follicles reached a diameter of 16–18 mm in order to achieve the final oocyte maturation.

The oocyte–cumulus complexes (COCs) were retrieved at ovum pick-up by transvaginal ultrasound guided puncture of follicles, approximately 34–36 h after the hCG administration.

### 2.4. Cumulus Cells Isolation

The procedure used for the CCs isolation from COCs is the same as previously described in our study [10]. Briefly, the removal of CCs from COCs was aided with the exposure of COCs to hyaluronidase solution (HYASE-10XTM, Vitrolife, Goteborg, Sweden). A subsequent mechanical repeatedly aspiration of the COCs in denudation pipettes completed the procedure of CCs isolation. At the end of the practice, the oocytes categorized as mature (nuclear metaphase II stage) were used for fertilization in vitro (ICSI procedure) and the relative CCs were stored at −80 °C until the use for the purpose of the present study. Of note that CCs deriving from metaphase II (MII) oocytes were pooled and collected separately for individual patients.

### 2.5. DNA, RNA and Protein Extraction

Collected pools of CCs were conserved in lysis buffer at −80 °C until the moment of manual nucleic acid and protein isolation. DNA, total RNA and proteins were extracted using the Nucleospin miRNA and RNA/DNA buffer set kits (Macherey-Nagel, Milan, Italy) according to the manufacturer’s instructions. Quantity and quality of DNA and total RNA were assessed by Qubit 2.0 (Invitrogen, Monza, Italy).

### 2.6. Telomere Length and Mitochondrial DNA Copy Number Measurement

Average TL and mtDNA-CN of CCs were evaluated with quantitative PCR (qPCR), using the Absolute Human Telomere Length and Mitochondrial DNA Copy Number Dual Quantification qPCR Assay Kit (AHDQ; ScienCell Research Laboratories, Carlsbad, CA, USA) following the manufacturer’s protocol. Telomere and mtDNA specific primers were used for the qPCR. A single copy reference (SCR) primer set was used as reference for data normalization. A reference genomic DNA sample with known TL and mtDNA-CN served as reference for calculating the TL and mtDNA-CN of the target samples. 32 cycles of 95 °C for 20 s, 52 °C for 20 s and 72 °C for 45 s PCR reactions were performed. All samples were tested in triplicate. A melting stage was added at the end of amplification. After the qPCR was done, statistical analysis was performed using the 2^−∆∆Ct^ method, considering *p*-values < 0.05 as significant.

### 2.7. Telomere Gene Expression Analysis

Expression analysis of *TERF1*, *TERF2* and *POT1* genes was performed by qRT-PCR. 1 μg of total RNA was used for cDNA synthesis through the high-capacity cDNA reverse transcription kit (Applied Biosystems, Foster City, CA, USA) under the following conditions: 25 °C for 10 min, 37 °C for 120 min, 85 °C for 5 min and cool at 4 °C. 96-well plates were used to carry out qRT-PCR analysis on the QuantStudio^TM^ 7 Pro Real-Time PCR detection system (Life Technologies, Carlsbad, CA, USA). Amplification reaction was prepared in a total volume of 20 μL containing Maxima SYBR Green/ROX qPCR Master Mix (2X) (Thermo Fisher Scientific, Waltham, MA, USA), 1 μL of 50 ng target cDNA and 0.3 μM of each primer. GAPDH was used as housekeeping gene. Each sample was run in triplicate. Specific primer pairs are reported in Table S1 of Supplementary Materials. The ΔΔCt method and t test were employed to assess the relative gene expression, considering data significant in smokers’ CCs versus control CCs when showing a fold change (fc) > 1.4 or <0.7 and a *p*-value < 0.05.

### 2.8. MicroRNA Target Analysis

Real-time quantification of microRNAs, hsa-miR-155-5p (MIMAT0000646; miRBase), hsa-miR-23a-3p (MIMAT0000078), and hsa-miR-185-5p (MIMAT0000455) was executed by highly sensitive stem-loop RT-PCR. MicroRNAs were reverted from 25 ng of total RNA by high-capacity cDNA reverse transcription kit (Applied Biosystems, Foster City, CA, USA), using 1 μM of specific stem-loop reverse primers (Table S2), designed with modifications to include the specific stem-loop structure and insure its stability, as follows: 16 °C for 30 min, followed by 60 cycles at 30 °C for 30 s, 42 °C for 30 s, and 50 °C for 1 s. Final incubation at 85 °C for 5 min was also added to inactivate the reverse transcriptase. cDNAs were stored at −20 °C until use. Amplification was carried out using SYBR Green, as previously described, and PCR-specific 1 µM Forward Primer for each microRNA, as well as 1 µM of Universal Reverse Primer (Table S2), respecting the following PCR cycling conditions: 95 °C for 10 min, followed by 40 cycles of 15 s at 95 °C and 40 s at 60 °C. Upon completion of the reaction cycles, melt curves were obtained by heating the reactions from 60 °C to 95 °C. The specificity of the primers was confirmed by the presence of a single peak in the melt curve generated for all miRNAs. Relative expression of small RNAs of interest, normalized to endogenous reference RNU44 (Table S2), was determined using the 2^−ΔΔCt^ method (*p*-value < 0.05).

### 2.9. Protein Target Analysis

The total cell lysates (50 µg) were used for electrophoresis and successively transferred to the polyvinylidenfluoride (PVDF) membranes. The membranes were first blocked with 5% of non-fat milk in PBS + 0.1% Tween-20 was kept for 2 h at room temperature and then incubated with different primary antibodies overnight at 4 °C. The primary antibodies used were anti-TRF1 antibody (4 µg/mL) (ab10579 Abcam, Cambridge, UK), anti -TRF-2 antibody (1:2000) (NB110-57130, Novus, Centennial, CO, USA), anti-POT1 Antibody (1:1000) (M1-P1H5, Novus) and anti-β-actin (1:750) (sc 69879, Santa Cruz Biotechnology, Dallas, TX, USA), the latter used as loading control. Then, the membranes were washed several times in PBS + 0.1% Tween-20. Subsequently, membranes were incubated with 1:5000 of peroxidase-conjugated goat anti-mouse secondary antibody (A90-116P, Bethyl Laboratories Inc., Montgomery, TX, USA) and peroxidase-conjugated goat anti-rabbit secondary antibody (A120-101P, Bethyl Laboratories Inc., Montgomery, TX, USA) for 1 h at room temperature. The enhanced chemiluminescence detection method (ECL) (Amersham Pharmacia Biotech, Milan, Italy) with photo documenter Alliance 2.7 (Uvitec, Cambridge, UK) were used to visualize the protein expression and the signals were analyzed through UVIband-1D gel analysis (Uvitec, Cambridge, UK) (Figure S1). Finally, the data were normalized with densitometric values obtained from the loading control β-actin. GraphPad Prism 5 (GraphPad, San Diego, CA, USA) software and t-test analysis were used for statistical significance. Values of *p* < 0.05 were considered statistically significant.

## 3. Results

### 3.1. Patients’ Characteristics

No statistically significant differences existed among the two populations of the study (smokers and non-smokers) in terms of demographic characteristics. The data are summarized in Table 1. In addition, no statistically significant differences were noted in terms of days of stimulation (9.4 ± 2.1 for non-smokers and 9.8 ± 0.8 for smokers) and total dose of gonadotropin administered (1260.0 ± 697.6 IU for non-smokers and 1695.0 ± 1336.8 IU for smokers). Comparable numbers of oocytes retrieved at ovum pick-up and metaphase II oocytes were found between the two study groups. In detail, the numbers of oocytes retrieved at ovum pick up were 11.6 ± 2.1 and 12.2 ± 8.9 for non-smokers and smokers, respectively, while 8.4 ± 1.9 and 8.8 ± 7.4 metaphase II oocytes (MII) were retrieved in non-smokers and smokers, respectively.

Concerning the smokers, the mean number of cigarettes smoked/daily was 8.2 ± 3.0. In addition, the mean total duration of exposure to cigarette smoke up to the moment of oocyte retrieval was 10.6 ± 1.5 years.

### 3.2. Decrease of Average Telomere Length and mtDNA Copy Number in Smokers’ CCs

**Figure 1:**
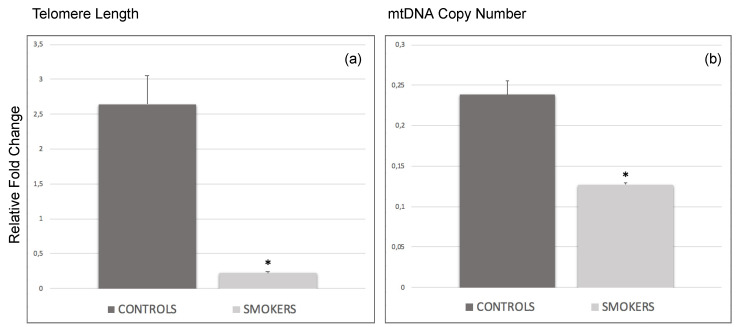
Dual quantification qPCR assay of average telomere length and mitochondrial DNA copy number in cumulus cells of smokers compared to corresponding controls. qPCR bars show the significant (* *p* < 0.05) relative fold changes ± SD of (**a**) average telomere length in CCs of smoking participants versus non-smoking controls and (**b**) average mtDNA copy number variation between smokers and corresponding age-matched controls.

Average TL and mtDNA-CN were analyzed in the CCs of the smoking and non-smoking participants, who served as age-matched controls, undergoing in vitro fertilization (IVF) treatments. Statistical analysis indicated a significantly lower relative TL (*p*-value = 0.014) and mtDNA-CN (*p*-value = 0.012) of the target sample to the reference sample in CCs of smokers compared to corresponding controls (Figure 1), indicating a possible correlation between chronic tobacco smoke exposure and aging-related conditions, as well as aberrant mitochondrial function in human female reproductive cells.

### 3.3. Down-Regulation of Genes Involved in Telomere Length and Protection

**Figure 2:**
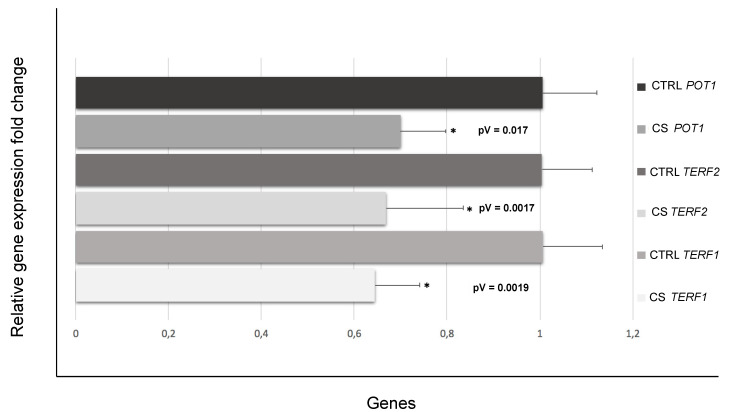
Significant mean relative fold change and *p*-values (pV) of down-regulated *TERF1*, *TERF2* and *POT1* genes in cumulus cells of female smokers (cigarette smoke (CS) *TERF1*, *TERF2* and *POT1*) versus age-matched controls (control (CTRL) *TERF1*, *TERF2* and *POT1*) * *p* < 0.05.

To better elucidate the underlying mechanisms between cigarette smoke exposure and the majorly accentuated telomere shortening in the CCs of smokers compared with those of non-smokers, the expressions of telomeric repeat binding factor 1 (*TERF1*), telomeric repeat binding factor 2 (*TERF2)* and protection of telomeres 1 *(POT1*) genes, actively involved in TL maintenance and protection, were additionally investigated. A significant down-regulation pattern (fold change < 0.7 and *p*-value < 0.05) was evidenced for all three described genes (Figure 2), showing potentially compromised TL integrity and telomere homeostasis.

### 3.4. Expression of TERF1, TERF2 and POT1 Protein Levels

**Figure 3:**
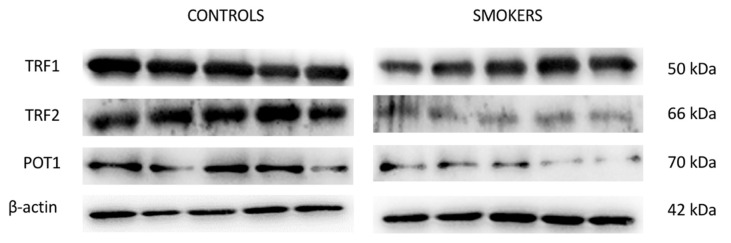
Western blotting analysis showing protein expression of TRF1, TRF2 and POT1 in the control and smokers’ group. To verify the loading consistency, the membranes were probed with β-actin. Western blot data shown are the representative data from three different experiments.

**Figure 4:**
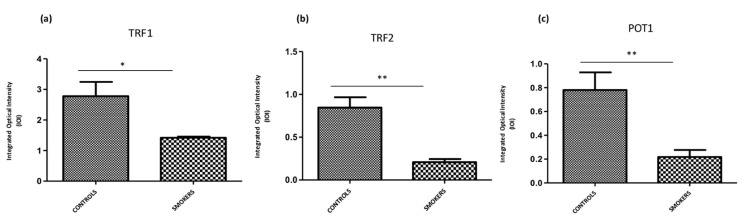
Histograms for TRF1 (**a**), TRF2 (**b**) and POT1 (**c**) represent densitometric measurements of proteins’ bands expressed as integrated optical intensity (IOI) mean of three separate experiments. The error bars show standard deviation (±SD). Densitometric values analyzed by *t*-test (unpaired *t*-test) return significant differences. ** *p* < 0.01, * *p* < 0.05.

To evaluate the level of expression of TERF1 (TRF1), TERF2 (TFR2) and POT1, Western blotting analysis was performed on the cumulus cell samples of control and smokers group. TERF1, TEFR2, and POT1 showed a significant down-expression in the CCs of smokers compared with the ones of the control group as demonstrated by protein-specific bands (Figure 3), and these results were confirmed by densitometric analyses (Figure 4).

### 3.5. Expression Profiling of miR-155, miR-23a and miR-185

The expression profiling analysis of the microRNAs has-miR-155-5hashsa-miR-23a-3phasnd hsa-miR-185-5p, correspondingly targeting genes *TERF1*, *TERF2*, and *POT1*, was performed in order to potentially underline a cause-related epigenetic mechanism behind the silencing of reported genes on behalf of these small non-coding RNAs. No significant differences were found in the miRNAs’ modulation in CCs of smokers compared with controls (one-way ANOVA).

## 4. Discussion

**Figure 5:**
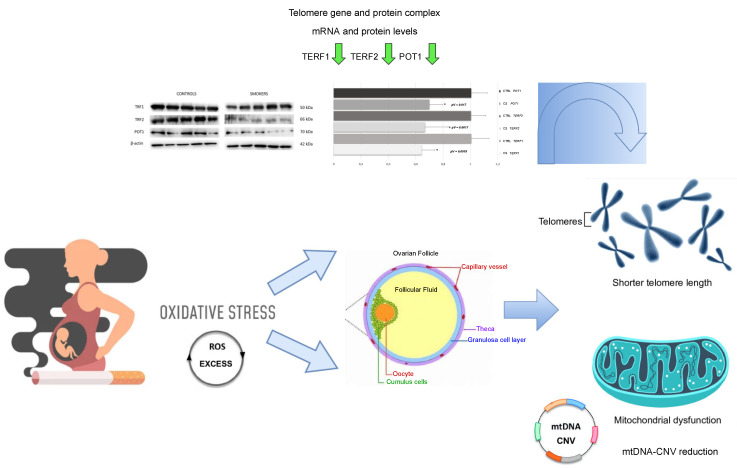
Correlation between cigarette smoke, mitochondrial DNA copy number variation (CNV), telomere shortening and related gene and protein expression due to suggested OS. * *p* < 0.05.

Smoking in women of reproductive age is known to severely impact the ovaries and reproductive cells such as oocytes [6] and CCs [10] and potentially carry a risk for premature menopause, premature birth, abnormal fetal growth, low birth weight, and miscarriage [17]. As we previously described [10], several differentially expressed key genes related primarily to OS were identified in CCs following chronic exposure to cigarette smoke, suggesting a lower antioxidant capacity in CCs of smoking versus non-smoking women. To better support our previous hypothesis, we investigated whether the already assumed smoking-derived OS in the CC samples of smoking women may cause cellular dysfunction altering well-known OS biomarkers such as TL, related genes, miRNAs, and protein expression, as well as mtDNA CNV. Although a cause-related effect between cigarette smoke-induced OS and telomere shortening and mtDNA CNV has been described in previous studies [11,14], the molecular mechanisms underlining this relationship in human CCs have still not been fully elucidated. To the best of our knowledge, this is the first study analyzing the relative TL and mtDNA-CN in human CCs to evaluate, at a molecular level, the damage triggered by cigarette smoking, presumably associated with alterations of OS. Following analysis of the data, we observed a significantly lower relative TL and mtDNA-CN in CCs of smokers compared to corresponding controls (Figure 1 and Figure 5).

Cigarette smoke can release multiple toxic compounds, generating OS characterized by the presence of superoxide anions and hydroxyl radicals [7]. Mitochondria are susceptible to this OS and are not able to remove or repair the reactive oxygen species (ROS)-induced DNA damage that was caused to them [18]. To compensate for this damage, healthy mitochondria tend to increase their DNA copy number in response to trans-acting factors that regulate, for instance, the replication and transcription of mtDNA, as well as the nuclear DNA-encoded mtRNA processing [19,20]. However, extensive OS may exceed mitochondrial capacity to make up for oxidative damage and reduce mtDNA content [18,21]. The observed mtDNA-CN reduction in our samples supports the idea that cigarette smoking impairs the health of CCs by modulating the expression of genes involved in protective cellular mechanisms and this may be associated with a decline in CCs’ terms of vitality, proliferation, and mitochondrial disfunction, not being able to mitigate the noxious effects of cigarette smoking. All these data are also in line with the observed telomere shortening in CCs of the smoking group of women analyzed in this study.

Telomeres consist of tandem arrays of TTAGGG sequence and serve as disposable DNA sequences that protect genomic DNA from shortening throughout replication [22]. Short or dysfunctional telomeres are perceived as DNA double-stranded breaks, causing cells to undergo premature replicative senescence [23]. TL is a complex hereditary trait, inevitably affected by aging and known to also be influenced by oxidative damage provoked by genetic, epigenetic, and environmental-related replicative stress. Furthermore, it is reportedly one of the most important causes of telomere shortening and reflects an imbalance between antioxidants and ROS [24,25]. Considering TL and fertility, the literature is abundant but also rather conflicting [26]. However, few data are currently available regarding specifically the correlation between TL in CCs and fertility and they do not consider the influence of cigarette smoking. Cheng et al. determined that the relative TL in CCs at the time of oocyte retrieval may be predictive of oocytes competence, as well as of good-quality embryos, but also affirmed that this may not be sufficiently discriminating in order to clinically be put into use and constitute a valid diagnostic tool [27]. In the study conducted by Yu et al., a possible connection between female age, cellular aging markers, aneuploidy rates in IVF protocols, and preimplantation genetic test for aneuploidy (PGT-A) cycles was taken into account. A negative correlation was established between TL in the granulosa cells and the aneuploidy rate in the young age group, emphasizing the need for PGT-A application in younger women [28]. Keefe et al. further hypothesized that ongoing shortening of telomeres, ranging from fetal oogenesis to the adult ovary, could be the cause of age-related ovarian impairment [29]. Moreover, shorter telomeres have also been found in leukocytes and granulosa cells in some cases of ovarian insufficiency [30]. On the other hand, Pedroso et al. did not detect any TL-related alteration in reproductive cells, such as cumulus cells of immature (CCI), cumulus cells of mature (CCM), germinal vesicle stage (GV) and MII oocytes of women diagnosed with polycystic ovary syndrome (PCOS) [31]. It has also been recently assumed that the maintenance system of TL may be atypical in the ovary. Both Morin et al. [32] and Lara-Molina et al. [33] discovered that TL is higher in CCs compared with peripheral blood leukocytes, indicating that the follicular environment could possess unusual mechanisms for dealing with telomere shortening.

To further suggest a mechanism by which presumed OS could possibly affect TL in CCs, we also quantified the mRNA levels and protein products of genes *TERF1*, *TERF2* and *POT1*, involved in the maintenance of telomeres. In these three transcripts, we detected a down-expression pattern in CCs of the smoking versus the non-smoking group. These results are in line with the observed telomere shortening, also known as telomere attrition, and aberrant telomere protection. In mammalian cells, telomere DNA is bound by Shelterin, a hexameric protein complex, which consists of TERF1, TERF2, RAP1, TIN2, POT1, and TPP1. The Shelterin complex suppresses an abnormal response to DNA damage and overall controls TL and DNA replication [34,35]. *TERF1* binds the double stranded TTAGGG repeats and is one of the key regulators of such telomere lengthening and replication of the DNA. At a cellular level, loss or reduction of TERF1 levels can result in telomeric DNA fragility and damage, as well as noticeable chromosomal instability and increase in telomere fusions [36,37]. In human cells, both *TERF1* and *TERF2* are predominantly located at chromosome ends where their main function is to contribute to the protection and maintenance of telomeric DNA [38]. The *TERF2* gene is involved in capping the chromosomes ends, thus downregulation in the expression of this particular gene may lead to broad cellular senescence-triggering telomere attrition. It has also been, consequently, suggested that shorter TL and downregulation of major Shelterin components, such as TERF2, can play a critical role in recurrent pregnancy loss by “telomere uncapping” [39]. TERF2 also protects chromosome ends against replicative DNA damage, especially those that take place due to significant topological stress [40]. Although TERF1 and TERF2 bind to the double-stranded telomeric DNA, POT1, on the other hand, binds the single-stranded overhang and interacts with the other Shelterin proteins via the linker proteins TIN2 and TPP1 [41]. *POT1* is involved in the DNA damage response and *POT1* deficiency has been found to potentially cause telomere instability and premature senescence [42]. In line with our gene expression analysis, Western Blotting results showed a down-expression of TERF1, TERF2 and POT1 also at a protein level, further validating telomere dysfunction and possible attrition in CCs of the smoking versus the control group in terms of translational regulation. These data confirm the important role of these proteins in telomere capping functions and trying to ensure a proper telomere maintenance as reported in literature [43]. In human endothelial cells, for instance, an intrinsic loss of telomere protection due to reduced TERF1 availability has been noticed due to biological aging [44]. TRF2 protein deficiency has also been found to potentially cause severe damage to telomere function and chromosomal instability, interfering with maintenance of normal cell physiological states in tumor formation [45]. Reduction of POT1 proteins levels can lead to loss of telomeric single-stranded overhangs and has also been associated with apoptosis, chromosomal instability, and senescence in cells [46] (Figure 5).

Finally, to have a complete view of the modifications due to cigarette smoking exposure, in this study we also analysed the expression of miRNAs involved in TL maintenance, namely miR-155, miR-23a and miR-185. Similarly to other cellular processes, genes involved in aging are known to be in some cases regulated by microRNAs. MicroRNAs (miRNAs) are short, non-coding RNAs that play a pivotal role in post-transcriptional regulation of gene expression [47]. Following stem-loop microRNA analysis, no statistically significant differences were found in the miRNAs’ modulation in CCs of smokers compared to corresponding controls, supporting the idea that this specific pathway is not involved in the regulation of gene expression modulated by cigarette smoking. Nonetheless, a much larger sample size would be able to detect statistically significant differences of smaller magnitude which may be relevant to confirm our data.

For a complete elaboration of the matter, it has been underlined, from a biological point of view, that even though several studies report that smoking in women of reproductive age is known to severely impact the ovaries and female reproductive system [6,10,17], the relationship between smoking and oocyte competence is inconclusive. Recently, for example, Bhide et al. did not find a statistically significant difference in quantitative ovarian reserve markers between current smokers, ex-smokers and never smokers [48]. Sansone et al. [49], on the other hand, aimed to analyze the interlink between the ovarian reserve decrease and lower oocyte quality derived by the follicle, connected to cigarette smoke. More specifically, the study focused on the possible impact of tobacco smoking on hormonal dosages in patients diagnosed with infertility, as well as patients subjected to recurrent miscarriages. No differences were detected between the two groups considered. The authors concluded that the association between cigarette smoke-contained nicotine and oocyte quality is a controversial research topic of significant importance.

Further studies are necessary to clarify the influence of nicotine on the ovarian reserve and identify the main risk factors. In this pilot study, we provided for the first time a detailed picture of molecular modulation by cigarette smoking in CCs, ranging from mtDNA-CNV and TL detection to miRNA and gene expression analysis of the TL maintenance-related pathway, as well as corresponding protein assessment.

## 5. Conclusions

Based on our previous findings [10], chronic tobacco smoke exposure was able to induce an overall down-regulation pattern in the expression of genes mostly involved in defense against OS in CCs of smoking women compared to non-smoking age-matched controls undergoing IVF, indicating a well-established overall diminished antioxidant capacity. In the current study, it was further highlighted that in CCs from the smoking group a decreased TL and mtDNA copy number, as well as downregulation of telomere maintenance and protection key genes and corresponding protein levels, were detected suggesting telomere dysfunction, chromosomal instability, and development of telomere attrition. Some limitations of the study should be recognized. First, the need for further large-scale studies regarding thoroughly established OS induced by smoking from a reproductive point of view and personalized antioxidant treatments aiming to enhance ART protocols in a clinical setup seems to be increasingly important. Secondly, TL and mtDNA CNV should also be analyzed in peripheral blood leukocytes to evaluate if they could mirror other somatic tissues as potential non-invasive biomarkers. Combined, our data add another piece to the puzzle of complex regulatory molecular networks controlling the tobacco smoke-associated CCs response where telomeres and mitochondria are co-regulated by multi-localization and multi-function proteins and RNAs.

## Figures and Tables

**Table 1:** Baseline characteristics of patients. AMH: anti-mullerian hormone, BMI: body mass index, FSH: follicle stimulating hormone. NS: Not significant.

	Non-Smokers (n = 5)	Smokers (n = 5)	*p*-Value
Age (years)	30.8 ± 0.8	33.2 ± 2.6	NS
AMH (ng/mL)	8.6 ± 2.5	9.2 ± 8.3	NS
Basal FSH (mIU/mL)	7.4 ± 3.7	6.8 ± 3.7	NS
BMI (kg/m^2^)	25.3 ± 4.5	24.6 ± 5.4	NS

## Data Availability

All original images and data are contained within the article.

## Supplementary Materials

The following supporting information can be downloaded at: https://www.mdpi.com/article/10.3390/antiox11091841/s1. Table S1: Real-Time PCR Primer Sequences and cycling conditions; Table S2: Stem-loop microRNA Primer Sequences; Figure S1: Full-size Western Blot membranes for all studied proteins.
