# Supplementary material for: The Aftermath of Long-Term Cigarette Smoking on Telomere Length and Mitochondrial DNA Copy Number in Human Cumulus Cells Prior to In Vitro Fertilization—A Pilot Study
Source: Antioxidants (Basel). 2022 Sep 19;11(9):1841. doi: 10.3390/antiox11091841 (PMC9495883; doi:10.3390/antiox11091841)
Supplement: Supplementary file 1 [file antioxidants-11-01841-s001.zip › antioxidants-1878387-supplementary.pdf]

Supplementary Materials

Table S1. Real-Time PCR Primer Sequences and cycling conditions

Primer to Analyze	TERF1<br>Gene Accession Number: NM_017489	TERF2<br>Gene Accession Number: NM_005652	POT1<br>Gene Accession Number:<br>NM_015450	GAPDH<br>Gene Accession Number:<br>NM_002046
Forward PCR Primer	5′- CCACATGATGGAGAAAATTAA GAGTTAT- 3′	5′- ACCAGGGCC TGTGGAAAAG- 3′	5′- CAGAACCTGACG ACAGCTTTCC- 3′	5′- AAGGTG AAGGTCGGA GTCAAC- 3′
Reverse PCR Primer	5′- TGCCGCTGCCTTCATTAGA- 3′	5′- GGTGGTTGGAGG ATT CCG TA-3′	5′- GCACATAGTGGT GTCCTCTCCA-3′	5′- GGGGTCATT GATGGCAAC AATA-3′
Annealing Temperature (°C)	60°C for 1 min			

Table S2. Stem-loop microRNA Primer Sequences

Name	MicroRNA Accession Number	Primer Sequences (5′–3′)
RT_miR-155	hsa-miR-155-5p (MIMAT0000646, miRBase)	GTTGGCTCTGGTGCAGGGTCCGAGGTATTCGCACCAGAGCCAACACCCCT
miR-155_Forward		GCGGCGGTTAATGCTAATCGTG
RT_miR-23a	hsa-miR-23a-3p (MIMAT0000078, miRBase)	GTTGGCTCTGGTGCAGGGTCCGAGGTATTCGCACCAGAGCCAACGGAAAT
miR-23a_Forward		GCGGCGGATCACATTGCCAGG
RT_miR-185	hsa-miR-185-5p (MIMAT0000455, miRBase)	GTTGGCTCTGGTGCAGGGTCCGAGGTATTCGCACCAGAGCCAACTCAGGA
miR-185_Forward		GCGGCGGTGGAGAGAAAGGCA
RT_RNU44	RNU44 (NR_002750, NCBI)	GTTGGCTCTGGTGCAGGGTCCGAGGTATTCGCACCAGAGCCAACAGTCAGTT
RNU44_Forward		GCGGCGGCCTGGATGATGATAG
Universal Reverse		GTGCAGGGTCCGAGGT

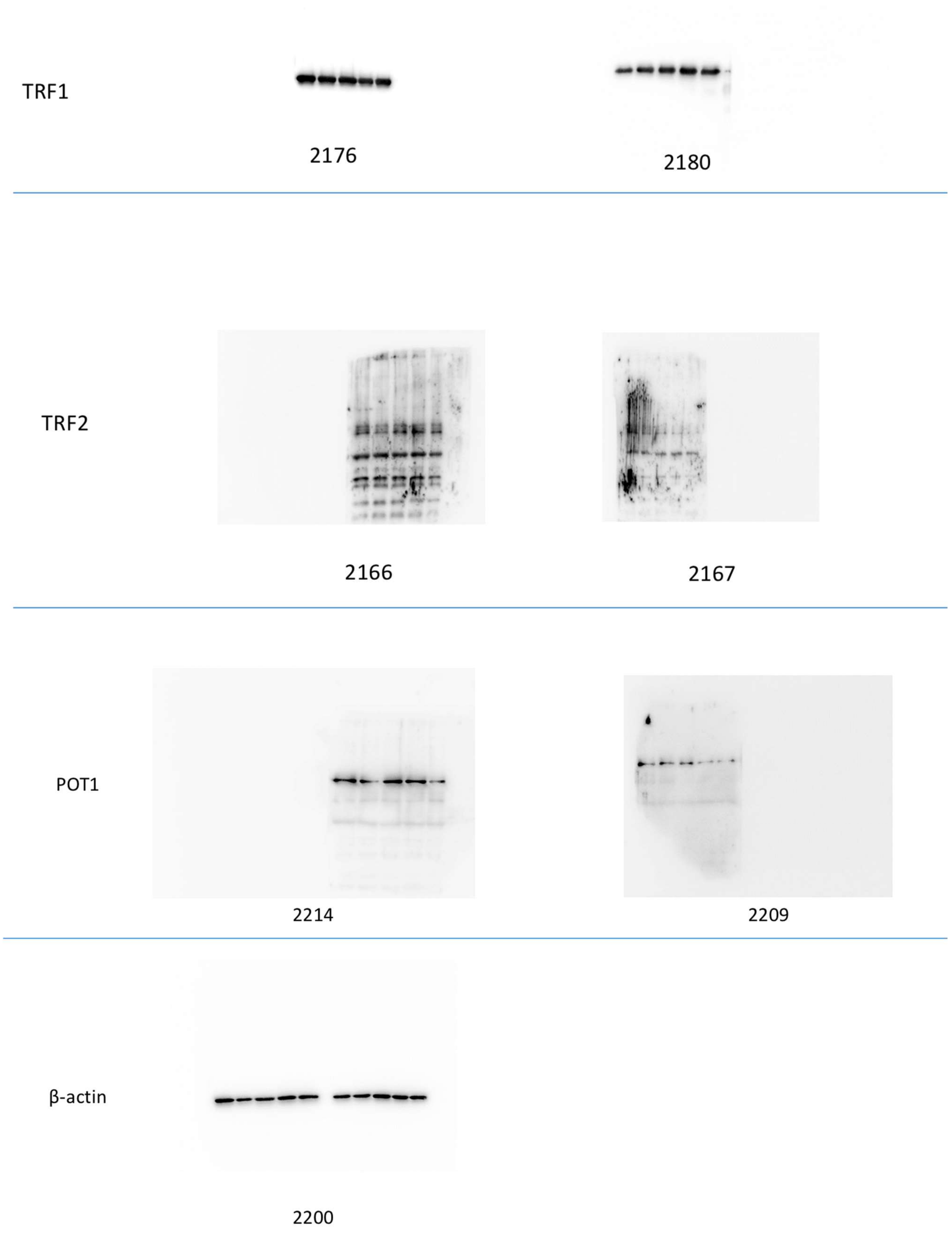

Figure S1. Full-size Western Blot membranes for all studied proteins
